# Oncologic Outcomes of Patients With Sarcomatoid Carcinoma of the Hypopharynx

**DOI:** 10.3389/fonc.2019.00950

**Published:** 2019-09-24

**Authors:** Liyuan Dai, Qigen Fang, Peng Li, Fei Liu, Xu Zhang

**Affiliations:** ^1^Department of Head Neck and Thyroid, Affiliated Cancer Hospital of Zhengzhou University, Henan Cancer Hospital, Zhengzhou, China; ^2^Department of Oral Medicine, The First Affiliated Hospital of Zhengzhou University, Henan Cancer Hospital, Zhengzhou, China

**Keywords:** sarcomatoid carcinoma, cancer of the hypopharynx, spindle cell carcinoma, head and neck spindle cell carcinoma, prognosis

## Abstract

**Objectives:** Sarcomatoid carcinoma (SaCa) of the hypopharynx is rare, and its clinical pathologic characteristics and prognosis remain unknown. Therefore, the study aimed to analyze the oncologic outcomes of patients with SaCa of the hypopharynx.

**Methods:** Patients with SaCa of the hypopharynx who were surgically treated in the period from January 1985 to December 2018 were enrolled from two clinical centers. A matched pair study was also performed, and each patient with SaCa of the hypopharynx was matched with one patient with squamous cell carcinoma (SCC) of the hypopharynx. The main study endpoint was disease-specific survival (DSS).

**Results:** A total of 62 patients (all male) were enrolled. Compared to patients with traditional SCC of the hypopharynx, patients with SaCa of the hypopharynx were older and had higher rates of perineural invasion, lymphovascular invasion and cancer cachexia. The 5-year DSS rate was 20% in patients with SaCa compared to 34% in patients in the matched group, and the difference was significant (*p* = 0.016). According to the univariate analysis, tumor stage, lymph node stage, disease stage, and cachexia were associated with DSS. According to the Cox model, neck lymph node metastasis and disease stage were independent predictors for worse DSS.

**Conclusion:** The prognosis of patients with SaCa of the hypopharynx is dismal, and this type of SaCa is associated with more aggressive biological behavior than traditional SCC of the hypopharynx; neck lymph neck node metastasis and disease stage were the most important predictors of DSS.

## Introduction

Since it was first reported by Virchow et al. (1) in 1864, sarcomatoid carcinoma (SaCa), a rare variant of squamous cell carcinoma (SCC), has been shown to be biphasic (2) and usually consists of both a conventional epithelial squamous cell component and a sarcomatous spindle cell component. Conventional SCC and sarcomatoid components have now been proven to arise monoclonally from a single stem cell (3–7). The disease is characterized by local recurrence and invasive growth, and is associated with poor prognosis. The tumor shows morphologic epithelial changes, where areas of squamous and spindle cell differentiation are observed (2–10).

The larynx is the most commonly involved site in SaCa of the head and neck region, followed by the oral cavity and hypopharynx (3). Cases of SaCa of the hypopharynx have only been described by a few authors (2, 3, 5–8, 11, 12); owing to the rarity of the disease, <50 cases have been reported, and detailed information regarding prognosis as well as prognostic factors for SaCa of the hypopharynx remain scarce. Therefore, we aimed to analyze the oncologic outcomes in SaCa of the hypopharynx by looking at outcomes occurring over a period of 30 years.

## Materials and Methods

The Zhengzhou University institutional research committee approved our study, all participants signed an informed consent agreement for medical research before initial treatment, and all experiments were performed in accordance with relevant guidelines and regulations.

**Figure 1:**
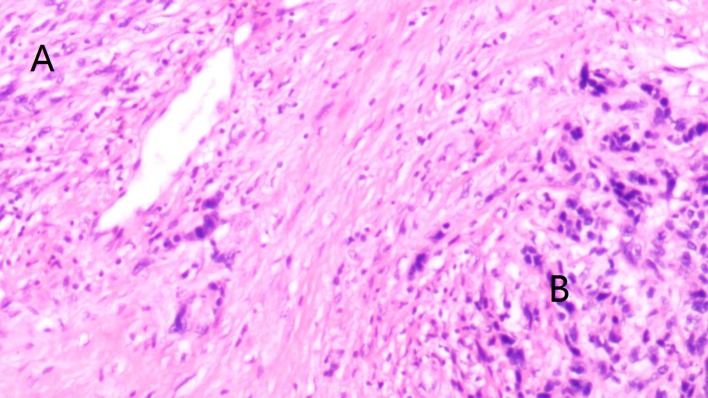
Accurate diagnosis of sarcomatoid carcinoma (SaCa) of the hypopharynx showing morphologic epithelial changes, where areas of spindle cell **(A)** and squamous **(B)** differentiation are demonstrated (hematoxylin and eosin stain × 20).

The medical records of patients with primary spindle cell carcinoma or SaCa of the hypopharynx who were surgically treated from January 1985 to December 2018 were reviewed from two hospitals: Affiliated Cancer Hospital and The First Affiliated Hospital of Zhengzhou University. The two clinical centers had the same treatment principle for hypopharynx malignancies. Information regarding age, sex, operation record, treatment, pathologic report, and follow-up was collected and analyzed. Patients were restaged by the 7th edition AJCC classification. Cancer cachexia was defined as an unintentional weight loss of at least 5% of the premorbid weight occurring over 3–6 months (13). All the pathologic sections were re-evaluated by at least two head and neck pathologists for the accurate diagnosis of SaCa of the hypopharynx (Figure 1). Perineural invasion was considered to be present if tumor cells were identified within the perineural space and/or nerve bundle, and lymphovascular infiltration was positive if a tumor was noted within the lymphovascular channels (14). Drinkers were defined as those who consumed at least one alcoholic drink per day for at least 1 year, and smokers were defined as those who smoked on a daily basis or who had quit smoking for <5 years (15).

To compare the survival rates between patients with SaCa of the hypopharynx and those with SCC of the hypopharynx, patients with SCC of the hypopharynx were also reviewed during the same study period in the two hospitals. Each patient with SaCa of the hypopharynx was matched with one patient with SCC of the hypopharynx, and the matching process was performed according to age, sex, perineural invasion, lymphovascular invasion, and disease stage (15).

The propensity score matching was used to perform the pair matching process. A Pearson chi-square test or Fisher's exact test was used to compare the clinical pathologic variables between the two groups. Disease-specific survival (DSS) was the main study endpoint, and it was calculated by the Kaplan-Meier method. The factors that were significant in the univariable analysis were then analyzed in the Cox model to determine the independent risk factors. All statistical analyses were performed using SPSS 20.0. A *p*-value of <0.05 was considered significant.

## Results

There were 62 (all male) patients with SaCa of the hypopharynx enrolled in total: 25 (40.3%) patients from the Affiliated Cancer Hospital and 37 (59.7%) patients from the First Affiliated Hospital. The mean age was 65.3 years old, with a range from 51 to 76, and smoking and drinking were noted in 41 (66.1%) and 29 (46.8%) patients, respectively. Cachexia was noted in 37 (59.7%) patients. Tumor stages were distinguished as T1 in 10 (16.1%) patients, T2 in 20 (32.2%) patients, T3 in 16 (25.8%) patients, and T4 in 16 (25.8%) patients. A total of 45 (72.6%) patients underwent partial pharyngolaryngectomy, and 17 (27.4%) patients underwent total pharyngolaryngectomy. Thirty-seven (59.7%) patients underwent primary closure, 15 patients (24.2%) underwent submental island flap reconstruction, and 10 patients (16.1%) underwent radial forearm flap reconstruction.

All patients underwent neck dissection, and a total of 59 selective neck dissections, 14 modified neck dissections, and five radical neck dissections were performed. Neck node metastasis was noted in 32 (51.6%) patients: N1 in 6 (18.8%, 6/32) patients, N2a in 10 (31.3%, 10/32) patients, N2b in 10 (31.3%, 10/32) patients, N2c in 3 (9.4%, 3/32) patients, and N3 in 3 (9.4%, 3/32) patients. The disease stages observed in patients were distinguished as I in 5 (8.1%) patients, II in 10 (16.1%) patients, III in 17 (27.4%) patients, and IV in 30 (48.4%) patients. Perineural invasion was reported in 23 (37.1%) patients, and lymphatic invasion was reported in 22 (35.5%) patients. A positive margin was demonstrated in 6 (9.7%) patients. Extracapsular extension of the node was reported in 17 (27.4%) patients. A total of 50 (80.6%) patients underwent postoperative radiotherapy, and 19 (30.6%) patients also received postoperative chemotherapy.

During the same period, there were 1,632 patients with traditional SCC of the hypopharynx. As described in Table 1, compared to traditional SCC of the hypopharynx, SaCa of the hypopharynx mainly affected male, older patients and was associated with a higher rate of perineural invasion; moreover, cancer cachexia was more common in patients with SaCa (all *p* < 0.05). No significant difference regarding tumor stage, neck lymph node stage, disease stage, lymphovascular invasion, or treatment was noted (all *p* > 0.05).

**Table 1:** Comparison of clinical pathologic variables between patients with sarcomatoid carcinoma (SaCa) and patients with traditional hypopharynx squamous cell carcinoma (SCC).

	**SaCa (*n* = 62)**	**SCC (*n* = 1,632)**	***p***
**Age**			
≥65	47 (75.8%)	1,002 (61.4%)	
<65	15 (24.2%)	630 (38.6%)	0.022
**Sex**			
Male	62 (100%)	1,248 (76.5%)	
Female	0	384 (23.5%)	<0.001
**Smokers**			
Yes	41 (66.1%)	956 (58.6%)	
No	21 (33.9%)	676 (41.4%)	0.236
**Drinkers**			
Yes	29 (46.8%)	589 (36.1%)	
No	33 (53.2%)	1,043 (63.9%)	0.086
**Cachexia**			
Yes	37 (59.7%)	745 (45.6%)	
No	25 (40.3%)	887 (54.4%)	0.030
**Tumor stage**			
T1 + T2	30 (48.4%)	749 (45.9%)	
T3 + T4	32 (51.6%)	883 (54.1%)	0.699
**Neck node stage**			
N0	30 (48.4%)	715 (43.8%)	
N+	32 (51.6%)	917 (56.2%)	0.476
**Disease stage**			
I + II	15 (24.2%)	519 (31.8%)	
III + IV	47 (75.8%)	1113 (68.2%)	0.206
**Perineural invasion^#^**			
Yes	23 (37.1%)	287 (24.0%)	
No	39 (62.9%)	908 (76.0%)	0.020
Lymphovascular invasion^*^			
Yes	22 (35.5%)	248 (20.3%)	
No	40 (64.5%)	971 (79.7%)	0.004
**Extracapsular spread^&^**			
Yes	17 (27.4%)	333 (24.9%)	
No	45 (72.6%)	1004 (75.1%)	0.655
**Treatment**			
Surgery	12 (19.4%)	245 (15.0%)	
Surgery + radiotherapy	31 (50.0%)	822 (50.4%)	
Surgery + radiotherapy +	19 (30.6%)	565 (34.6%)	0.601
chemotherapy			

# *Status of perineural invasion in 437 patients with traditional SCC was unknown*.

* *Status of lymphovascular invasion in 413 patients with traditional SCC was unknown*.

& *Status of extracapsular spread in 295 patients with traditional SCC was unknown*.

*Gray value indicates significant variables*.

**Figure 2:**
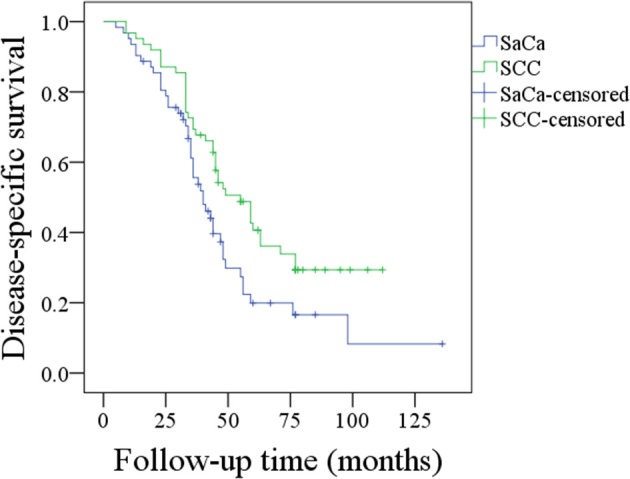
Comparison of disease-specific survival between patients with sarcomatoid carcinoma (SaCa) of the hypopharynx and patients matched for squamous cell carcinoma (SCC) (*p* = 0.016).

During our follow-up period with a mean time of 40.1 (range: 5–136) months, recurrence was noted in 49 (79.0%) patients: 22 (44.9%, 22/49) were cases of local recurrence, 14 (28.6%, 14/49) were cases of regional recurrence, 7 (14.3%, 7/49) were cases of loco-regional recurrence, and 6 (12.2%, 6/49) were cases of distant recurrence. Fifteen (30.6%, 15/49) patients experienced recurrence that was successfully salvaged by surgical treatment: 8 (53.3%, 8/15) patients underwent radical neck dissection, 5 (33.3%, 5/15) patients underwent total pharyngolaryngectomy, and 2 (13.3%, 2/15) patients underwent both radical neck dissection and total pharyngolaryngectomy. Forty-four (71.0%) patients died of the disease, and the 5- and 10-year DSS rates were 20 and 8%, respectively. After being matched, the two groups had no significant difference regarding age, sex, tumor stage, neck lymph node stage, disease stage, perineural invasion, lymphovascular invasion, or treatment (Table 2, all *p* > 0.05). In matched patients, the 5- and 10-year DSS rates were 34 and 28%, respectively, and the difference was significant (*p* = 0.016) (Figure 2).

**Table 2:** Comparison of clinical pathologic variables between patients with sarcomatoid carcinoma (SaCa) and matched patients with traditional hypopharynx squamous cell carcinoma (SCC).

	**SaCa (*n* = 62)**	**SCC (*n* = 62)**	***p*^#^**
**Age**			
≥65	47 (75.8%)	47 (75.8%)	
1<65	15 (24.2%)	15 (24.2%)	NS
**Sex**			
Male	62 (100%)	62 (100%)	
Female	0	0	NS
**Smokers**			
Yes	41 (66.1%)	37 (59.7%)	
No	21 (33.9%)	25 (40.3%)	NS
**Drinkers**			
Yes	29 (46.8%)	33 (53.2%)	
No	33 (53.2%)	29 (46.8%)	NS
**Cachexia**			
Yes	37 (59.7%)	37 (59.7%)	
No	25 (40.3%)	25 (40.3%)	NS
**Tumor stage**			
T1 + T2	30 (48.4%)	27 (43.5%)	
T3 + T4	32 (51.6%)	35 (56.5%)	NS
**Neck lymph node stage**			
N0	30 (48.4%)	34 (54.8%)	
N+	32 (51.6%)	28 (45.2%)	NS
**Disease stage**			
I + II	15 (24.2%)	15 (24.2%)	
III + IV	47 (75.8%)	47 (75.8%)	NS
**Perineural invasion**			
Yes	23 (37.1%)	23 (37.1%)	
No	39 (62.9%)	39 (62.9%)	NS
**Lymphovascular invasion**			
Yes	22 (35.5%)	22 (35.5%)	
No	40 (64.5%)	40 (64.5%)	NS
**Extracapsular spread**			
Yes	17 (27.4%)	14 (22.6%)	
No	45 (72.6%)	48 (77.4%)	NS
**Treatment**			
Surgery	12 (19.4%)	11 (17.7%)	
Surgery + radiotherapy	31 (50.0%)	33 (53.2%)	
Surgery + radiotherapy +	19 (30.6%)	18 (29.0%)	NS
chemotherapy			

# *NS, not significant*.

With regard to the survival analysis in patients with SaCa, tumor stage, neck lymph node stage, disease stage, cachexia, and extracapsular extension were associated with the DSS rate according to the univariable analysis, and further Cox model analysis described that neck node stage and disease stage were the independent predictors for DSS rate (Table 3).

**Table 3:** Univariable and cox model analysis of predictors for the disease-specific survival in patients with hypopharynx sarcomatoid carcinoma.

**Variables**	**Univariable**	**Cox model**	
	***p***	***p***	**Exp(B) (95% CI)**
Age (<65 vs. ≥65)	0.185		
Smoke	0.385		
Alcohol	0.560		
Tumor stage (T1 + T2 vs. T3 + T4)	0.040	0.277	1.680 (0.402–7.011)
Node stage (N0 vs. N+)	<0.001	0.048	1.339 (1.011–4.697)
Disease stage (I+ II vs.!!break III + IV)	0.013	0.002	2.090 (1.231–6.913)
Perineural invasion	0.175		
Lymphovascular invasion	0.403		
Cachexia	0.035	0.611	0.821 (0.287–4.341)
Extracapsular extension	0.021	0.058	1.339 (0.968–2.998)
Radiotherapy	0.689		
Chemotherapy	0.738		
Margin status	0.366		

*Gray value indicates significant variables*.

## Discussion

Since SaCa was first reported in 1864 (1), a few studies have described its clinical and pathologic characteristics in the throat, the parotid gland and other subsites of the head and neck region (2–12). However, due to the rarity of the disease, <50 cases of SaCa of the hypopharynx have been previously reported (2–12), and the prognosis and prognostic factors remain unknown in SaCa of the hypopharynx; the current study was the first to present the clinical results of SaCa of the hypopharynx. SCC is the most common pathologic type found in head and neck cancer (16). Previous evidence has shown an evolution from conventional epithelial cancer to SaCa as well as a malignant nature of the sarcomatoid component (7). There might be variations in survival between SaCa and conventional SCC. Our matched analysis supports the hypothesis that a significantly lower DSS rate is noted in patients with SaCa than in patients with SCC, and a similar finding was also reported by Chang et al. (8) in SaCa of the head and neck. However, it is prudent to conclude that the prognosis of SaCa of the hypopharynx is poorer than that of SCC of the hypopharynx: first, both studies had a limited sample size (62 cases and 18 cases), and second, even if the matching process had taken several confounding factors into consideration, the different radiation techniques and margin statuses were not analyzed between the two groups, owing to the long time span; these factors are typically independent negative prognostic predictors in head and neck cancer (17).

The detailed clinical pathologic characteristics of SaCa of the hypopharynx remain unclear. Gamez et al. (4) reported that the majority of patients with SaCa of the larynx were elderly males who presented with early-stage disease. Berthelet et al. (3) also reported a male prevalence and a low risk of lymph node metastasis in SaCa of the head and neck. Our findings partially supported these viewpoints: all patients were male, and compared to patients with traditional SCC, patients with SaCa were older. However, hoarseness was apparent even in every patient with early-stage SCC of the larynx; however, there are typically no obvious symptoms that appear until advanced-stage cancer of the hypopharynx occurs, which might explain why most of our patients presented with advanced-stage disease. Cancer cachexia is a common concomitant phenomenon in SCC of the head and neck, and approximately one-third of patients have cachexia at diagnosis (18, 19). This finding is partially reflected by the degree of tumor malignancy (20); data demonstrate a higher degree of tumor malignancy in patients with SaCa than in our patients with traditional SCC of the hypopharynx and in previous reports (18, 19). Additionally, perineural invasion and lymphovascular invasion are two of the most important prognostic factors in SCC of the head and neck, and it has been noted that there are higher rates of perineural invasion and lymphovascular invasion in patients with SaCa. These findings suggest that the biological behavior of SaCa is more aggressive and might result in poorer survival than that of SCC.

The prognosis of patients with SaCa of the head and neck is typically dismal. In a report by Berthelet et al. (3), a total of 17 patients were enrolled for analysis, and the authors described that the median survival time was 32 months, with an actual survival of 72 and 42% at 2 and 5 years, respectively. In another paper focusing on outcomes in SaCa of the larynx, the authors reported that the 5-year overall survival, progression-free survival, and local control rates were 63, 46, and 72%, respectively (4). This finding apparently conflicts with our findings. The 5-year DSS rate was found to be only 20% in the current study; the first possible explanation for this finding is that advanced stage disease predominated in the current study, unlike in the abovementioned studies. Similarly, in a report published by Chang et al. (8), of the 78 included patients, 64% (50) were classified as having T3 or T4 tumors at the time of diagnosis, and the 5-year survival rate was 14%. Second, all the cases of disease in the current study involved localization in the hypopharynx, and cancer of the hypopharynx is usually associated with a poorer prognosis than cancers in other subsites of the head and neck (21).

Overall lymph node metastasis is typically uncommon in SaCa of the head and neck. Gamez et al. (4) reported that positive neck disease represented only 5% of the cases of SaCa of the larynx, and Niu et al. (10) reported that 5 (20%) patients had pathologic node metastasis out of the 15 patients undergoing neck dissection. However, in the current study, it was noted that 32 (51.6%) patients had neck metastatic disease, and this striking difference might be associated with the anatomic characteristics of the hypopharynx. There are abundant lymphatic vessels in the hypopharynx, and movements related to swallowing and speech can promote the occurrence of metastasis. Moreover, routine neck dissection was included in the primary treatment strategy in the two cancer centers, but in the abovementioned studies, elective neck dissection was more commonly performed, and this discrepancy might mask the true incidence of metastasis.

Prognostic factors have been evaluated for SaCa of the head and neck. Chang et al. (8) found that in a surgical intervention group, only a history of previous SCC produced good outcomes. The practice of neck dissection, the use of adjuvant therapies, or cancer stage did not affect the overall survival rate. Niu et al. described that in SaCa of the parotid gland, perineural nerve invasion was the only independent predictor for DSS (10). In the current study, neck lymph node metastasis was significantly associated with the DSS rate in SaCa of the hypopharynx. A higher neck lymph node metastasis stage and disease stage were associated with a higher risk of death, and a similar finding was also noted in SCC of the head and neck (22, 23).

The effectiveness of radiotherapy and chemotherapy for increasing local-regional and distant control in SCC of the head and neck has been widely accepted, but there is little evidence in the literature on the role of such adjuvant treatments for the treatment of SaCa. In a report by Chang et al. (8), 41 (64.1%) of the patients received radiotherapy, and the author found that no apparent survival benefit was associated with radiotherapy. In another paper focusing on SaCa of the larynx (24), the authors reported that compared to a 57.1% DSS rate with nonsurgical treatment, surgery led to a 5-year DSS rate of 84.1%, and adjuvant radiotherapy was not advised. Similarly, we also failed to note that postoperative adjuvant treatment could improve DSS. However, owing to the difference in the lymph node metastasis rate and pattern between cancer of the larynx and hypopharynx, the application of adjuvant radiotherapy cannot be easily excluded, and more studies are needed to clarify this question.

In summary, the prognosis of patients with SaCa of the hypopharynx is dismal, and SaCa of the hypopharynx is associated with more aggressive biological behavior than traditional SCC of the hypopharynx; neck lymph node stage and disease stage were the most important predictors for DSS.

## Data Availability Statement

All datasets generated for this study are included in the manuscript/supplementary files.

## Ethics Statement

The Zhengzhou University institutional research committee approved our study and all participants signed an informed consent agreement for medical research before initial treatment. And all the related procedures were consistent with Ethics Committee regulations.

## Author Contributions

All authors contributed to data analysis, drafting, and revising the article, gave final approval of the version to be published, and agree to be accountable for all aspects of the work.

### Conflict of Interest

The authors declare that the research was conducted in the absence of any commercial or financial relationships that could be construed as a potential conflict of interest.
